# Ingramon-Depot Therapy in db/db Mice: The Impact on Local and Systemic Inflammation and Myocardial Remodeling

**DOI:** 10.3390/biomedicines14102341

**Published:** 2026-10-14

**Authors:** Anastasiia Yu. Filatova, Natalya V. Radyukhina, Andrei M. Karhov, Ella V. Kurilina, Natalya Yu. Ruleva, Anastasia E. Yanina, Ekaterina S. Ogurtsova, Vladislav S. Kuzmin, Maria V. Sidorova, Tatiana I. Arefieva

**Affiliations:** 1 Laboratory of Cellular Immunology, National Medical Research Center of Cardiology Named After Academician E.I. Chazov, Academician Chazov St., 15a, 121552 Moscow, Russia; nradukhina@mail.ru (N.V.R.); nruleva@mail.ru (N.Y.R.); oghurtsova20@mail.ru (E.S.O.); tiarefieva@cardio.ru (T.I.A.); 2 Scientific Research Support Resource Center, National Medical Research Center of Cardiology Named After Academician E.I. Chazov, Academician Chazov St., 15a, 121552 Moscow, Russia; 3 Laboratory of Cardiac Electrophysiology, National Medical Research Center of Cardiology Named After Academician E.I. Chazov, Academician Chazov St., 15a, 121552 Moscow, Russia; akarchoff@gmail.com (A.M.K.); ku290381@mail.ru (V.S.K.); 4 Pathology Department, National Medical Research Center of Cardiology Named After Academician E.I. Chazov, Academician Chazov St., 15a, 121552 Moscow, Russia; evkurilina@cardio.ru; 5 Faculty of Medicine, Lomonosov Moscow State University, Lomonosovsky Prospekt, 27/1, 117192 Moscow, Russia; ianina2025ana@gmail.com; 6 Department of Human and Animal Physiology, Lomonosov Moscow State University, Leninskiye Gory, 1, 12, 119234 Moscow, Russia; 7 Laboratory of Peptide Synthesis, National Medical Research Center of Cardiology Named After Academician E.I. Chazov, Academician Chazov St., 15a, 121552 Moscow, Russia; mvsidorova@cardio.ru

**Keywords:** myocardial fibrosis, db/db mice, diabetic cardiomyopathy, heart failure, Ingramon

## Abstract

**Introduction**: Chronic systemic inflammation is a major driver of myocardial fibrosis in patients with metabolic syndrome; accordingly, db/db mice with impaired leptin signaling are widely used to model myocardial injury associated with obesity and hyperglycemia. This study investigates the impact of the peptide Ingramon, an antagonist of MCP-1 and interleukin-8, on systemic and local inflammation and myocardial remodeling in db/db mice. **Methods**: Sixteen-week-old female db/db mice were assigned to an experimental group that received subcutaneous injections of Ingramon encapsulated in PLGA microcapsules (Ingramon-depot; 30 mg/kg; n = 13) once every 2 weeks for 16 weeks or to a control group that received empty microcapsules as a placebo (n = 11). Transthoracic echocardiography was performed before and after treatment. At the end of the experiment, the mice were euthanized, and blood, adipose tissue, and heart tissue samples were collected for further analysis. **Results**: Ingramon-depot administration was associated with changes in adipose tissue leukocyte composition. The proportions of F4/80-positive macrophages tended to be lower, and the macrophage-to-T-cell ratio was significantly reduced. Morphological analysis showed significantly less cardiomyocyte granular degeneration/coagulative necrosis and myocardial fibrosis in Ingramon-depot-treated mice. Transcriptomic analysis of the myocardium revealed lower expression of neutrophil granule genes at the gene-set level in Ingramon-depot-treated mice. **Conclusions**: These findings suggest that Ingramon may exert cardioprotective effects in a murine model of diabetic cardiomyopathy; the underlying mechanisms, including a possible impact on inflammatory-cell recruitment, require further investigation.

## 1. Introduction

The increasing global burden of cardiometabolic disease, particularly cardiovascular disease, obesity, and diabetes, highlights the need to better define the mechanisms by which metabolic dysfunction promotes adverse myocardial remodeling. Moreover, in recent decades, obesity and diabetes have become highly prevalent noncommunicable diseases worldwide [1,2]. In addition, patients with diabetes mellitus exhibit a significantly elevated risk of cardiovascular events and all-cause mortality in comparison with non-diabetic populations [1,2]. Obesity is of particular relevance, since its associated cardiometabolic disturbances—including visceral adiposity, impaired metabolic flexibility, and chronic low-grade inflammation—contribute to pathological cardiac remodeling and progressive myocardial dysfunction [3].

Metabolically active adipose tissue is increasingly recognized as a critical source of adipokines and cytokines that initiate and sustain so-called meta-inflammation. This chronic low-grade inflammatory milieu, in turn, may promote maladaptive pathological alterations in multiple organs, including the heart [4]. Adipose tissue macrophages are key mediators of meta-inflammation, as demonstrated in both experimental and clinical studies [5,6]. Elevated extracellular glucose concentration, adipose-tissue chemokine production, and protein glycation with subsequent alarmin formation promote macrophage accumulation and their transformation into a pro-inflammatory M1 phenotype [7]. As a consequence, myocardial fibrosis emerges as a clinically important manifestation of persistent cardiometabolic inflammation and plays a central role in the onset and progression of heart failure (HF).

A key early event mediating myocardial fibrosis is the increased production of chemotactic cytokines by myocardial cells, including monocyte chemoattractant protein-1 (MCP-1), which facilitates recruitment of circulating monocytes. Previous studies using low-density lipoprotein receptor-deficient (Ldlr−/−) mice fed a Western-type diet demonstrated epigenetic reprogramming of granulocyte–macrophage progenitors, resulting in the differentiation of hyperinflammatory monocytes [8]. These cells may be recruited to the myocardium and other organs, where they can amplify inflammatory responses and promote tissue injury. The inflammatory microenvironment of the injured heart also attracts neutrophils, which exacerbate myocardial injury by enhancing pro-inflammatory signaling, generating reactive oxygen species, and releasing proteolytic enzymes that remodel the extracellular matrix. Thus, myocardial fibrosis is associated with infiltration by activated T-lymphocytes and neutrophils, M1 macrophage polarization, and increased secretion of pro-inflammatory cytokines, including interleukin(IL)-1β, IL-6, tumor necrosis factor (TNF), and transforming growth factor-β, as well as chemokines such as CCL2, CXCL12 [9,10]. Taken together, the available data support the hypothesis that systemic immune cell activation in obesity, including monocytes and neutrophils, contributes substantially to their myocardial accumulation. Thus, a major current challenge is the development of effective anti-inflammatory strategies or anti-inflammatory treatments capable of reducing cardiovascular risk in individuals with obesity and diabetes.

Db/db mice are considered one of the appropriate models for studying cardiometabolic disorders. In these mice, a mutation in the gene encoding the long isoform of the leptin receptor impairs leptin signaling in the hypothalamus, leading to hyperphagia and obesity [11]. At 6–8 weeks of age, db/db mice develop obesity-associated hyperglycemia and progressive insulin resistance due to obesity [12]. In addition to hyperglycemia/ insulin resistance, their phenotype includes metabolic abnormalities such as dyslipidemia together with arterial hypertension, which contributes to the development of left ventricular (LV) hypertrophy and diastolic dysfunction. Enlargement of the left atrium can already be observed in adolescent db/db mice [13,14]. Histological analysis of the myocardial tissue reveals hypertrophied cardiomyocytes, as well as prominent interstitial and perivascular fibrosis accompanied by an increased collagen content [15,16]. Thus, the db/db murine model reproduces both functional and histological features of human HF associated with metabolic disorders and obesity [16].

Despite its clinical relevance, anti-inflammatory therapeutic strategies aimed at preventing myocardial fibrosis remain insufficiently investigated, and inhibitors of monocyte and neutrophilic granulocyte chemotaxis have not been systematically evaluated. Early studies in animal models of myocardial hypertrophy showed that suppression of MCP-1 activity using an anti-MCP-1-neutralizing monoclonal antibody ameliorated fibrosis in the LV myocardium and improved diastolic function [17,18]. Inhibition of MCP-1 activity in CCR2- [19] or CCL2/MCP-1-deficient mice also prevented LV remodeling and HF development [18]. We previously described Ingramon, an anti-inflammatory 12-member peptide corresponding to residues 65–76 of human MCP-1. Ingramon acts as an MCP-1 antagonist by displacing the chemokine from glycosaminoglycan-binding sites [20], thereby preventing the formation of the concentration gradient required for directional cell migration. Ingramon also antagonizes IL-8, the principal neutrophil chemoattractant. Compared with chemical compounds, viral proteins, and antibodies, peptide-based drugs are generally associated with low allergenicity, favorable tolerability, and safety during repeated administration. Preclinical and clinical studies have demonstrated the safety and efficacy of Ingramon in animals and humans [20]. Encapsulation of Ingramon in biodegradable poly(lactic-co-glycolic acid) (PLGA) microcapsules, which have high biocompatibility and generally cause only a minimal, transient reaction [21], produces a prolonged-release formulation (Ingramon-depot) suitable for outpatient treatment [22]. In the mouse “air pouch” model, we observed suppression of CCL2/JE-stimulated monocyte migration into the “pouch” 7 days after intramuscular administration of Ingramon-depot, supporting its prolonged effect [22].

The present study investigated the effects of Ingramon-depot on systemic and local inflammation, as well as on myocardial remodeling, in db/db mice.

## 2. Materials and Methods

All experimental procedures were designed and conducted in accordance with accepted ethical standards for animal research and complied with the “European Convention for the Protection of Vertebrate Animals Used for Experimental and Other Scientific Purposes” (Strasbourg, 18 March 1986).

### 2.1. Animals

Female C57BLKS/J homozygous db/db mice were obtained from the SPF-Laboratory of the Institute of Cytology and Genetics, Siberian branch of the Russian Academy of Sciences, while female C57BL/6 mice were obtained from FSBI Scientific Center of Biomedical Technologies FMBA of Russia. Animals were housed in the Bioclinic of FSBI National Medical Research Centre of Cardiology named after Academician E.I. Chazov under standard conditions, with regular rodent chow and water available ad libitum and a 12 h light/dark cycle.

### 2.2. Experimental Design

**Figure 1:**
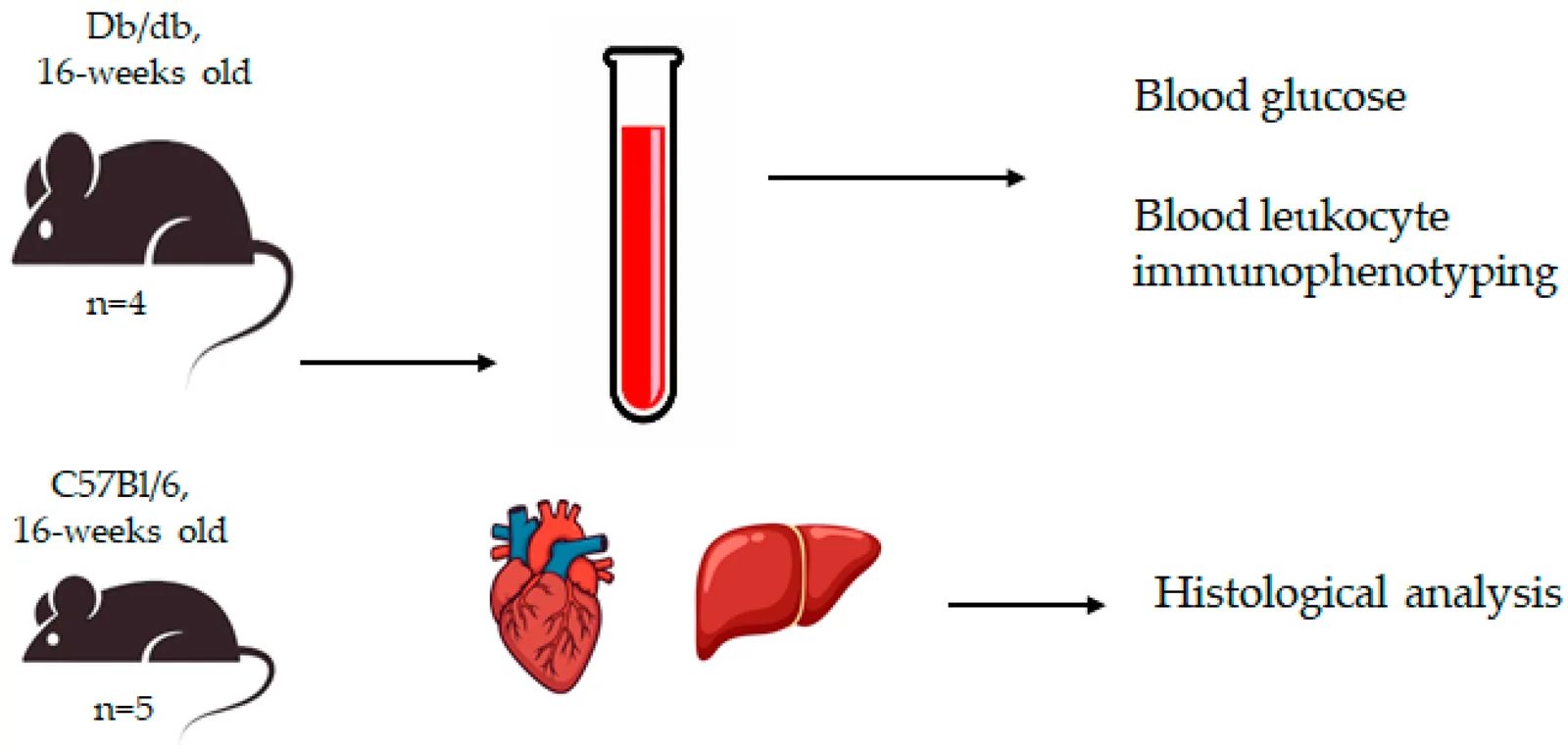
Experimental design for experiment 1.

In experiment 1, 16-week-old db/db (n = 4) and C57BL/6 (n = 5) mice were sacrificed to compare blood glucose concentrations and leukocyte composition, and heart and liver tissues were subsequently collected for further morphological analysis (Figure 1).

**Figure 2:**
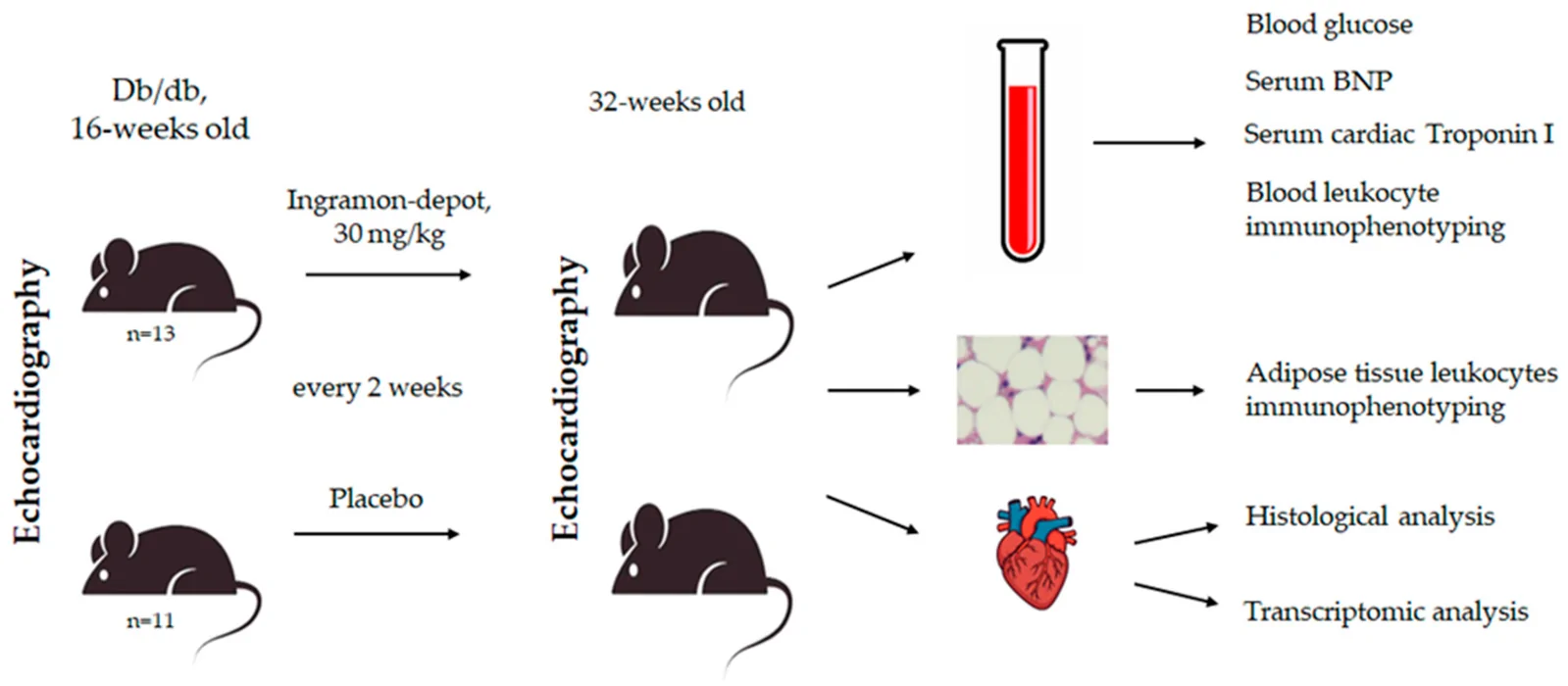
Experimental design for experiment 2.

In experiment 2, 16-week-old female db/db mice received subcutaneous injections once every 2 weeks for 16 weeks of either Ingramon-depot (prepared as described previously [22]) at a dose of 30 mg/kg in a mannitol solution, corresponding to 1200 µg per mouse (experimental group, n = 13), or placebo, consisting of empty PLGA microcapsules suspended in mannitol solution (control group, n = 11). The dose was selected based on previous studies demonstrating peptide efficacy at doses ranging from 40 µg/kg to 40 mg/kg without observed toxic effects and on the release kinetics of Ingramon from the microcapsules [22,23,24,25]. Transthoracic echocardiography was performed at 16 and 32 weeks of age, after which the animals were sacrificed and blood, adipose tissue, and heart samples were collected for further analyses, as summarized in Figure 2.

### 2.3. Echocardiography

Transthoracic echocardiography was performed using a Visualsonics Vevo1100 ultrasound in vivo imaging system equipped with an MS400 linear array transducer (FUJIFILM VisualSonics, Toronto, ON, Canada). The mice were anesthetized using 3–4% isoflurane for induction and 1–2% for measurements. The examination and measurements were conducted in a blinded manner. Standard B-mode (2D) images of the heart and pulsed Doppler images of the mitral valve inflow were acquired to assess cardiac dimensional and functional parameters, as described [26]. In end diastole, the septum wall thickness (SWTd), posterior LV wall thickness (PWTd), and end-diastolic and end-systolic internal diameter of the LV (LVIDd, LVIDs) were measured on the 2D echo recordings. Fractional shortening (FS) was calculated by dividing the difference between LVIDd and LVIDs by the LVIDd. Relative wall thickness (RWT) was calculated by dividing the sum of SWTd and PWTd by the LVIDd. The mass of the LV myocardium (LVMM) was assessed using the following equation: 1.05 × ([SWTd + LVIDd + PWTd]3 − [LVIDd]3] [27]. LV end-diastolic volume (EDV) and end-systolic volume (ESV) were measured using the Teichholz formula: EDV = 7 × LVIDd3/(2.4 + LVIDd); ESV = 7 × LVIDs3/(2.4 + LVIDs). LV ejection fraction (LVEF) was determined as (EDV − ESV)/EDV × 100. LV diastolic function was assessed by measuring pulsed Doppler mitral peak E-wave and A-wave velocities (in early and late diastole, respectively). Myocardial performance index (Tei index) was derived using conventional pulsed Doppler echocardiography and determined by dividing the sum of isovolumetric relaxation time (IVRT) and isovolumetric contraction time (IVCT) by the aortic ejection time [28]. All echocardiographic measurements were averaged over at least 3 cardiac cycles and performed according to current guidelines for cardiac physiological assessment in mice [29].

### 2.4. Blood Leukocytes

Blood samples, 500–1000 mL, were collected rapidly from the LV after isoflurane inhalation and cervical dislocation using a syringe containing EDTA at a 6:1 ratio. Complete peripheral blood counts were analyzed using a Sysmex XN-1000 hematology analyzer (Sysmex Corporation, Kobe, Japan) and verified visually with a hemocytometer.

### 2.5. Biochemical Analysis

Blood glucose concentrations were measured using an iChek glucose analyzer (DIAMEDICAL Ltd., London, UK).

### 2.6. Enzyme Immunoassay (ELISA)

Blood samples were allowed to clot at room temperature for 30 min before centrifugation at 3000× *g* for 10 min, after which serum samples were collected. Serum concentrations of brain natriuretic peptide (BNP) and cardiac troponin I were measured by an enzyme-linked immunosorbent assay (ELISA; Cloud-Clone Corp., Wuhan, China) according to the manufacturer’s instructions.

### 2.7. Blood and Adipose Tissue Leukocyte Immunophenotyping

Whole blood was collected from the right ventricle in an EDTA-anticoagulated Vacutainer tube. The samples were processed within 1 h after being collected. Adipose tissue leukocyte cells were isolated from subcutaneous adipose tissue, as described previously [30]. Suspensions of adipose tissue leukocytes and whole blood were stained with appropriate antibodies to surface antigens. The following antibodies and reagents were used: CD45-PerCP, F4/80-FITC, CD206-APC, Ly6G-PE, CD3-PE, CD4-FITC, CD25-PE, CD8-PercP, CD115-PC5 (Elabscience Biotechnology, Wuhan, China, Caltag Laboratories, Carlsbad, CA, USA) and lysing and fixing solutions (BD Biosciences, San Jose, CA, USA). Stained samples were analyzed with a two-laser FACS Calibur flow cytometer with CellQuest Pro software version 6.0(Becton Dickinson Immunocytometry Systems, San Jose, CA, USA) and Northern Lights with SpectroFlo version 3.2.1 (Cytek (Wuxi) Biosciences Co., Ltd, Wuxi, China). Lymphocytes were gated according to light scatter characteristics and CD45 expression pattern, monocytes were identified as CD115-positive cells, and neutrophils were defined as Ly6G-positive cells (Figures S1 and S2A, Supplement). Macrophages were identified as F4/80-positive cells, and CD206 expression was used to distinguish between M1 (F4/80+/CD206−) and M2 (F4/80+/CD206+) macrophage subpopulations (Figure S2B, Supplement). Among CD3-positive T cells, CD4-positive helper T cells, CD4+CD25+ regulatory T cells, and CD8-positive cytotoxic T cells were quantified (Figure S1, Supplement).

### 2.8. Histology

After isoflurane inhalation and cervical dislocation, mice were sacrificed, and the hearts and livers were excised. Transverse myocardial slices measuring 1–1.5 mm were obtained and fixed in 4% formaldehyde solution. Paraffin-embedded heart sections of 1–2 µm thickness were stained with hematoxylin–eosin (H&E), periodic acid-Schiff (PAS), and Masson trichrome (MT). MT- and H&E-stained sections were digitized using an Aperio CS2 digital slide scanner (Leica Biosystems Imaging, Inc., San Diego, CA, USA) at the Scientific Research Support Resource Center of the National Medical Research Center of Cardiology named after Academician E.I. Chazov. Fibrosis was quantified in MT-stained sections by analyzing the entire LV areas using Aperio ImageScope software (version 12.4.6, Leica Microsystems, Wetzlar, Germany). Fibrotic areas were identified using the Positive Pixel Count 2004-08-11 algorithm. Fibrosis was quantified as the ratio of the positive pixels to the total number of pixels within the LV. In H&E-stained sections, areas of degeneration/necrosis were annotated throughout the LV myocardium of each mouse and expressed as the ratio of the annotated area to the total LV area. Additional high-magnification examinations of H&E- and PAS-stained sections were performed using a CTR5000 microscope (Leica, Mannheim, Germany). The investigator analyzed sections in a blinded manner.

### 2.9. Transcriptomic Profiling

Three pooled samples per group, each comprising heart apex tissue from 3–4 mice, were used for mRNA sequencing analysis. Libraries were prepared using the RNA-seq Library Prep Kit for Illumina (Vazyme, Nanjing, China). Library quality control was carried out using capillary electrophoresis (Qsep400, High Sensitivity Cartridge Kit, Bioptic, Taiwan, China). Library concentration was measured using the SpectraQ HS (Raissol, Moscow, Russia). Sequencing was performed on a SURFSeq 5000 platform using the SURFSeq 5000 Sequencing Kit V1.0 FCH 300 cycles (GeneMind, Shenzhen, China).

The reads were aligned to the M. musculus genome using STAR software (version 2.7.11b). As a reference genome and annotation information for genes, fasta and gtf GRCm39 (GCA_000001635.9) files obtained from the Ensembl database were used. Sequencing was performed using paired-end sequencing, providing an average sequencing depth of 39.05 ± 3.01 million reads per sample. Quantitative values (raw gene-level read counts) for each gene were determined based on alignment results using FeatureCounts software (Subread package, version 2.1.1; Walter and Eliza Hall Institute of Medical Research, Melbourne, Australia). Normalization was performed using the median-of-ratios method implemented in DESeq2. TPMs were evaluated afterwards. Per-sample sequencing depth and read-assignment metrics are given in Table S1 (Supplement).

Differential expression analysis was performed with DESeq2 using the design ~ group (Ingramon-depot versus placebo) and median-of-ratios normalization. For the exploratory gene list, protein-coding genes with a nominal *p* < 0.05 and |log2 fold change| > 1 were selected. As these *p*-values are not corrected for multiple testing, Benjamini–Hochberg adjusted *p*-values are additionally reported for all tested genes (Table S2, Supplement), and genes with an adjusted *p* < 0.05 were considered significant after correction.

Pre-ranked gene set enrichment analysis was performed with GSEApy v1.3.1 (pre-rank module, 1000 permutations) on all tested genes ranked by the DESeq2 Wald statistic. Reactome and Hallmark gene sets for mouse were taken from MSigDB v2026.1. Sets containing 15–500 genes were tested, and those with FDR < 0.05 were considered enriched (Table S3). Robustness was assessed by repeating the analysis after exclusion of each animal in turn, with genes ranked by the difference in mean log2 normalized counts.

### 2.10. Statistics

Sample size calculation. For experiment 2, the sample size was calculated a priori using data from Tu Y. et al. [31], who reported myocardial collagen areas of 0.98 ± 0.39% in untreated db/db mice and 0.59 ± 0.19% in treated db/db mice (mean ± SD; picrosirius red), corresponding to Cohen’s d = 1.3. With a two-sided *t*-test, α = 0.05 and 80% power, 11 animals per group were required. To allow for attrition, 13 animals per group were enrolled. Two control animals died, resulting in final group sizes of 11 control and 13 treated animals, which gives 86% power. The calculation was performed using G*Power version 3.1.9.7 (Heinrich-Heine-Universität Düsseldorf, Düsseldorf, Germany). Experiment 1 was exploratory and included five animals per group; one db/db mouse died before 16 weeks of age.

Statistical analysis was performed using MedCalc software, version 20.104 (MedCalc Software Ltd., Ostend, Belgium). The continuous data are presented as a median (25th, 75th percentile). The categorical variables are presented as counts and percentages. The Mann–Whitney U test was used to compare quantitative non-normally distributed variables. The categorical variables were assessed using the Fisher’s exact two-tailed test for paired comparisons, respectively. Differences were considered statistically significant at *p* < 0.05.

## 3. Results

### 3.1. Experiment 1

To assess blood leukocyte composition, heart function using echocardiography, and myocardial tissue morphology in db/db diabetic mice, 16-week-old db/db mice were compared with age-matched C57BL/6 controls. At this age, db/db mice exhibited marked obesity, with body weight approximately twice that of C57BL/6 mice (49.0 (41.5; 53.0) g vs. 25.0 (24.7; 26.1) g, respectively, *p* = 0.014) and significantly higher blood glucose concentrations (47.1 (34.1; 48.8) mmol/L vs. 8.7 (7.4; 9.0) mmol/L, respectively, *p* = 0.0009).

Significant differences in peripheral blood leukocyte composition were observed between wild-type (C57BL/6) and db/db mice, indicating the presence of chronic systemic inflammation in the latter. Compared with C57BL/6 controls, db/db mice showed increased numbers of neutrophilic granulocytes and monocytes, whereas lymphocyte counts, including CD4+ and CD8+ T-cell subsets, were significantly reduced (Table 1).

Echocardiographic assessment demonstrated preserved LV contractile function and no signs of LV hypertrophy in 16-week-old db/db mice (Table 2). Structural cardiac parameters did not differ significantly between the db/db and C57BL/6 mice; however, functional assessment revealed an approximately two-fold lower transmitral E/A ratio in db/db mice, suggesting impaired LV relaxation from an early age.

**Figure 3:**
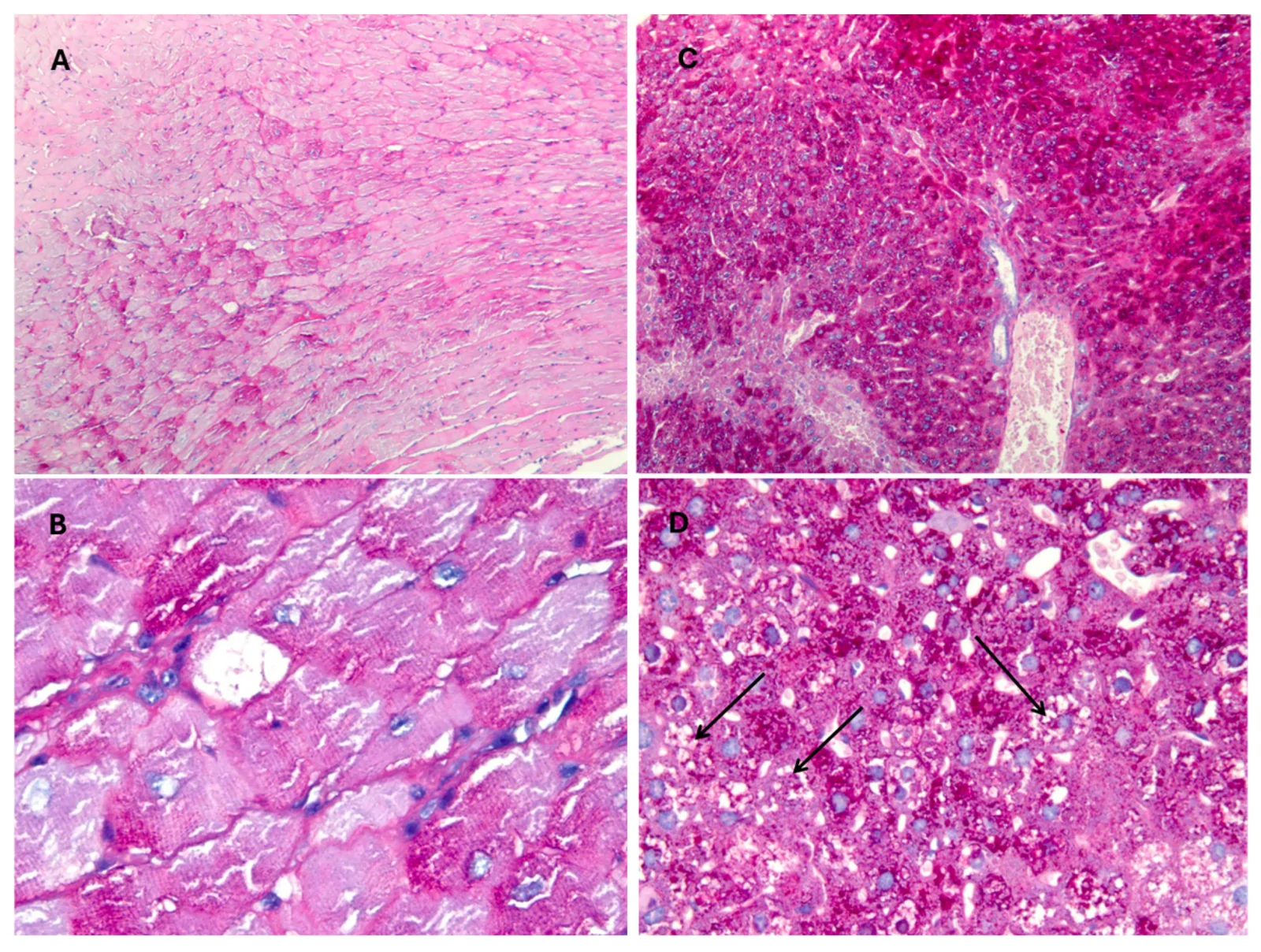
Glycogen deposits in cardiomyocytes ((**A**) (×100) and (**B**) (×630)) and hepatocytes ((**C**) (×100) and (**D**) (×400)) of db/db mice, PAS staining. Hepatocytes with vacuolar (fatty) degeneration are marked by arrows.

Histopathological examination revealed characteristic diabetic cardiomyopathy alterations in 16-week-old db/db mice. PAS staining demonstrated pronounced glycogen accumulation in cardiomyocytes, whereas only small, scattered glycogen granules are typically observed in the cardiomyocytes of healthy mice. In contrast to the C57BL/6 controls, db/db mice also showed hepatocellular vacuolar, or fatty, degeneration and glycogen-overloaded hepatocytes (Figure 3).

**Figure 4:**
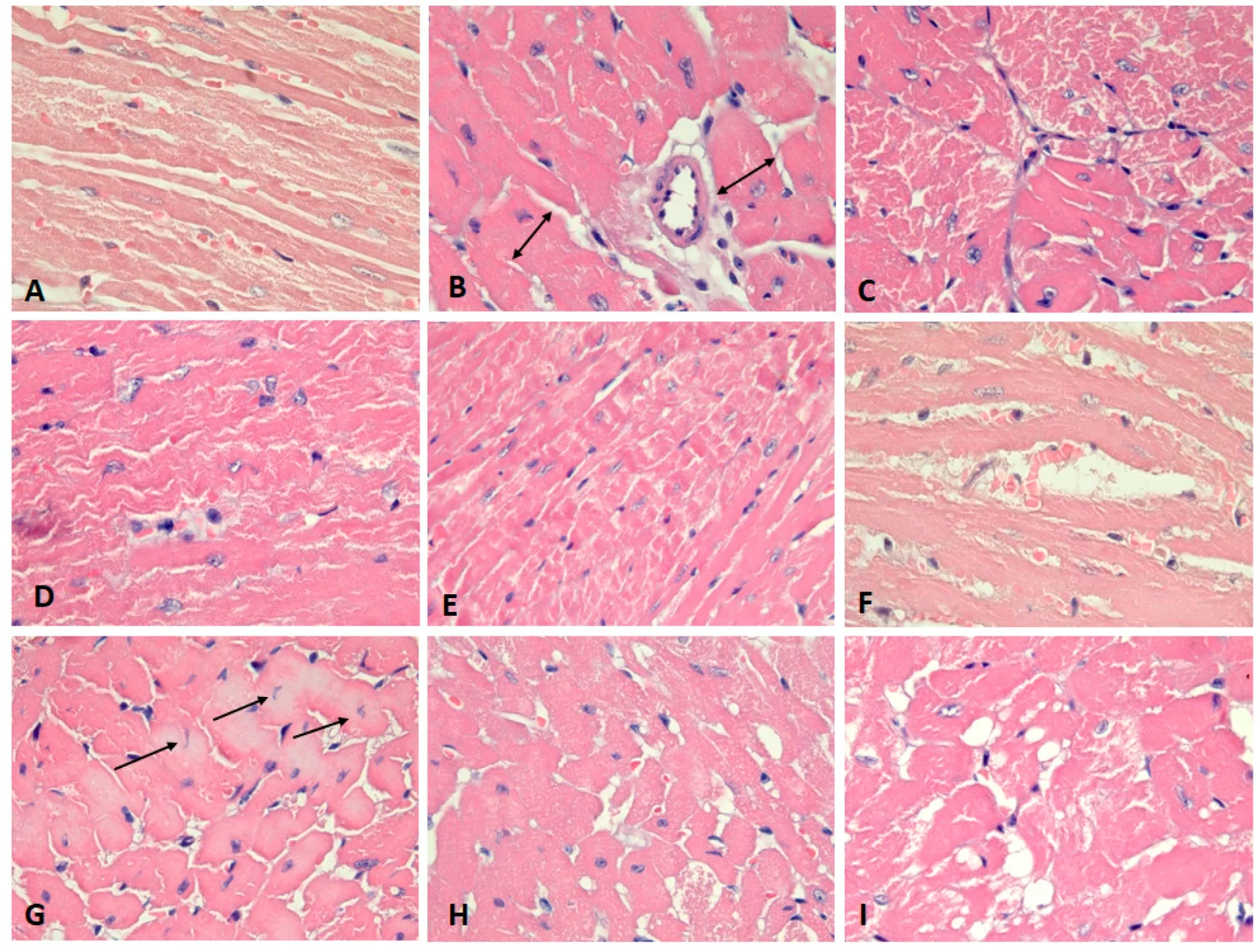
Representative pathomorphological alterations in the myocardium of 16-week-old db/db mice (H&E staining, ×630). (**A**) cardiomyocyte dystrophy, granular degeneration, interstitial edema; (**B**) arteriole wall segmental hyalinization and perivascular edema. Blood elements are visible adhering to the hyalinized vessel wall. Focus of hypertrophied cardiomyocytes (arrows indicate the diameters of the enlarged cells); (**C**) foci of coagulative necrosis, the formation of fibrous tissue; (**D**) wave-like deformation of cardiomyocytes; (**E**) contraction bands; (**F**) erythrostasis and “sludge” phenomenon in the capillary; (**G**) cytoplasmic hyalinization, nuclear karyolysis (arrows indicate the nuclei); (**H**) microvacuolar (fatty) degeneration; (**I**) fatty degeneration.

Morphological examination of the LV myocardium further confirmed pathological alterations consistent with diabetic cardiomyopathy. These included myocardial interstitial and perivascular edema, pronounced signs of cardiomyocyte granular degeneration, mild interstitial and perivascular fibrosis, areas of undulating cardiomyocyte deformation, and contraction bands. Db/db mice exhibited hyalinization of myocardial vessel walls and leukocyte adhesion in small vessels associated with a risk of thrombosis, while erythrocyte stasis and aggregation were observed within microcirculation. At the cellular level, marked cardiomyocyte dystrophy was evident, including granular degeneration/coagulative necrosis, vacuolar (fatty) degeneration, and hyaline degeneration, and nuclear changes such as karyopyknosis, karyorrhexis, and karyolysis were observed. Hypertrophic and dystrophic cardiomyocytes were also detected (Figure 4), whereas no pathological changes were observed in healthy C57BL/6 mice, whose LV morphology corresponded to expected age-related findings (the data are not presented).

Overall, 16-week-old db/db mice exhibited diabetes-related alterations, including obesity, hyperglycemia, and glycogen accumulation in cardiomyocytes and hepatocytes. These changes were accompanied by chronic systemic inflammation and marked LV myocardial injury at both the tissue and cellular levels, consistent with diabetic cardiomyopathy.

### 3.2. Experiment 2

The effects of 16 weeks of Ingramon-depot treatment were evaluated in db/db mice, according to the experimental design shown in Figure 2. Hyperglycemia was present in both the Ingramon-depot and placebo groups (47.1 (34.2; 65.0) mmol/L versus 45.7 (31.0; 48.5) mmol/L, respectively; *p* = 0.624). Ingramon-depot administration was associated with a non-significant reduction in blood BNP concentration compared with placebo (44.0 (32.1; 57.1) pg/mL vs. 62.0 (44.0; 82.1) pg/mL, respectively, *p* = 0.401). Blood cardiac troponin I concentrations did not differ significantly between mice treated with Ingramon-depot and those receiving placebo (32.4 (12.0; 33.7) pg/mL vs. 29.1 (11.7; 37.4) pg/mL, respectively, *p* = 0.784). Blood leukocyte immunophenotyping revealed no significant differences between db/db mice treated with Ingramon-depot and those receiving placebo (Table 3).

Immunophenotyping of adipose tissue leukocytes showed a tendency toward lower proportions of F4/80 macrophages in Ingramon-depot-treated mice. This tendency was observed in macrophages with both high and low CD206 expression (Table 4). Ingramon-depot administration was also associated with an increased percentage of CD3+ T-cells, whereas the proportion of neutrophilic granulocytes remained unchanged.

To evaluate cardiac structural and functional changes, transthoracic echocardiography was performed in 32-week-old db/db mice treated with Ingramon-depot or placebo (Table 5). Echocardiographic assessment showed no significant difference between the two groups. Ingramon-depot treatment also had no effect on mouse body weight (45.5 (25.5; 48.5) g vs. 45.5 (25.5; 48.5) g, respectively; *p* = 0.873) or heart weight (137.0 (125.0; 147.0) mg vs. 135.5 (127.0; 140.0) mg, respectively; *p* = 0.808).

**Figure 5:**
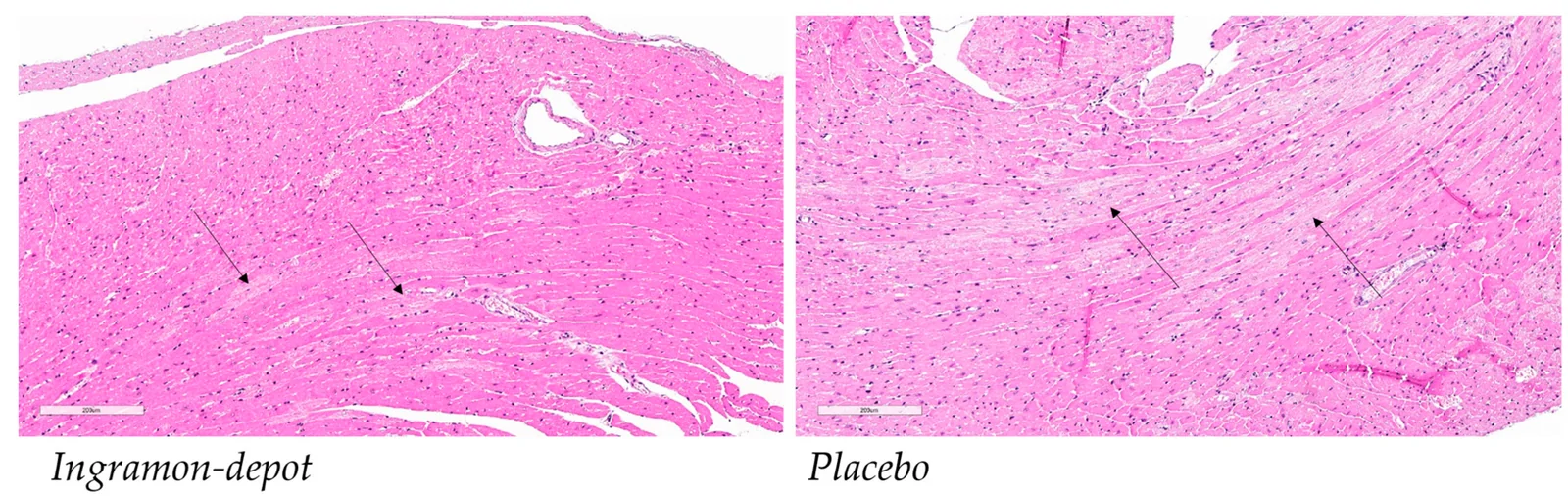
Representative LV myocardium histological tissue sections showing areas of granular degeneration/coagulative necrosis (marked by arrows) in db/db mice treated with Ingramon-depot or placebo (H&E staining, scale bar 200 μm).

**Figure 6:**
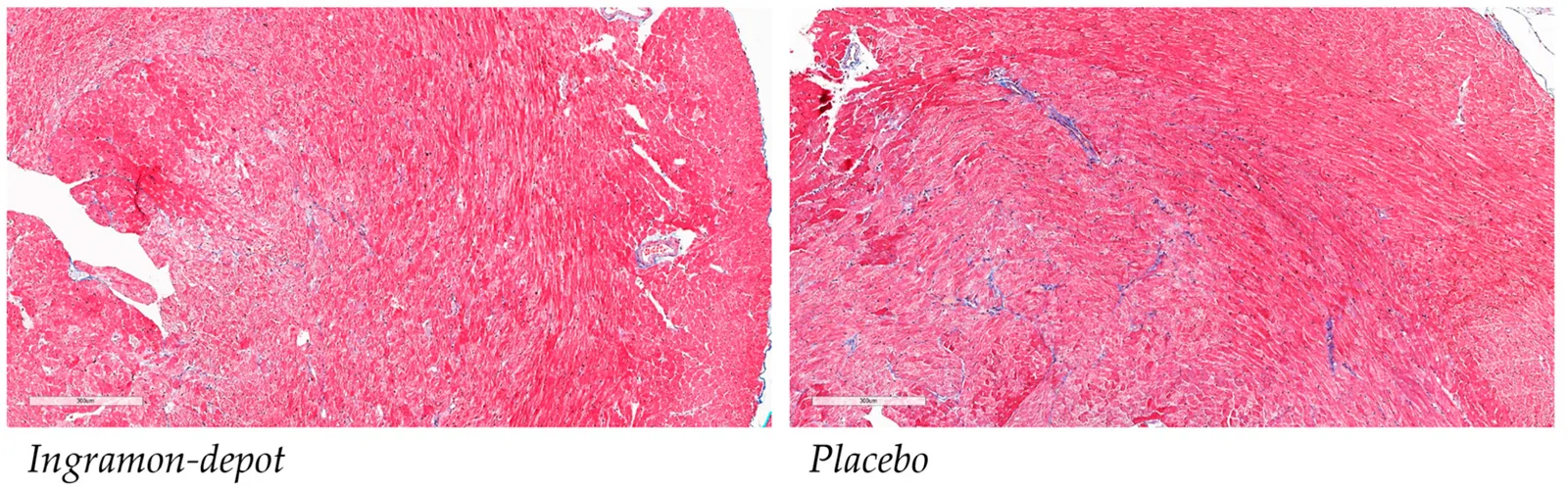
Representative LV myocardium histological tissue sections showing myocardial fibrosis (blue color) in db/db mice treated with Ingramon-depot or placebo (TM staining, scale bar 300 μm).

Subsequent between-group morphological analysis revealed significant differences in the fraction of cells with signs of granular degeneration/necrosis and fibrotic remodeling. Compared with placebo, Ingramon-depot treatment reduced the percentage of cardiomyocytes showing signs of granular degeneration/coagulative necrosis (45.0 (30.0; 60.0) % vs. 30.0 (6.3; 47.0) %, respectively, *p* = 0.048) and decreased myocardial fibrosis (5.3 (4.4; 6.6) % vs. 3.7 (2.8; 4.1) %, respectively; *p* = 0.0007) (Figure 5 and Figure 6).

**Figure 7:**
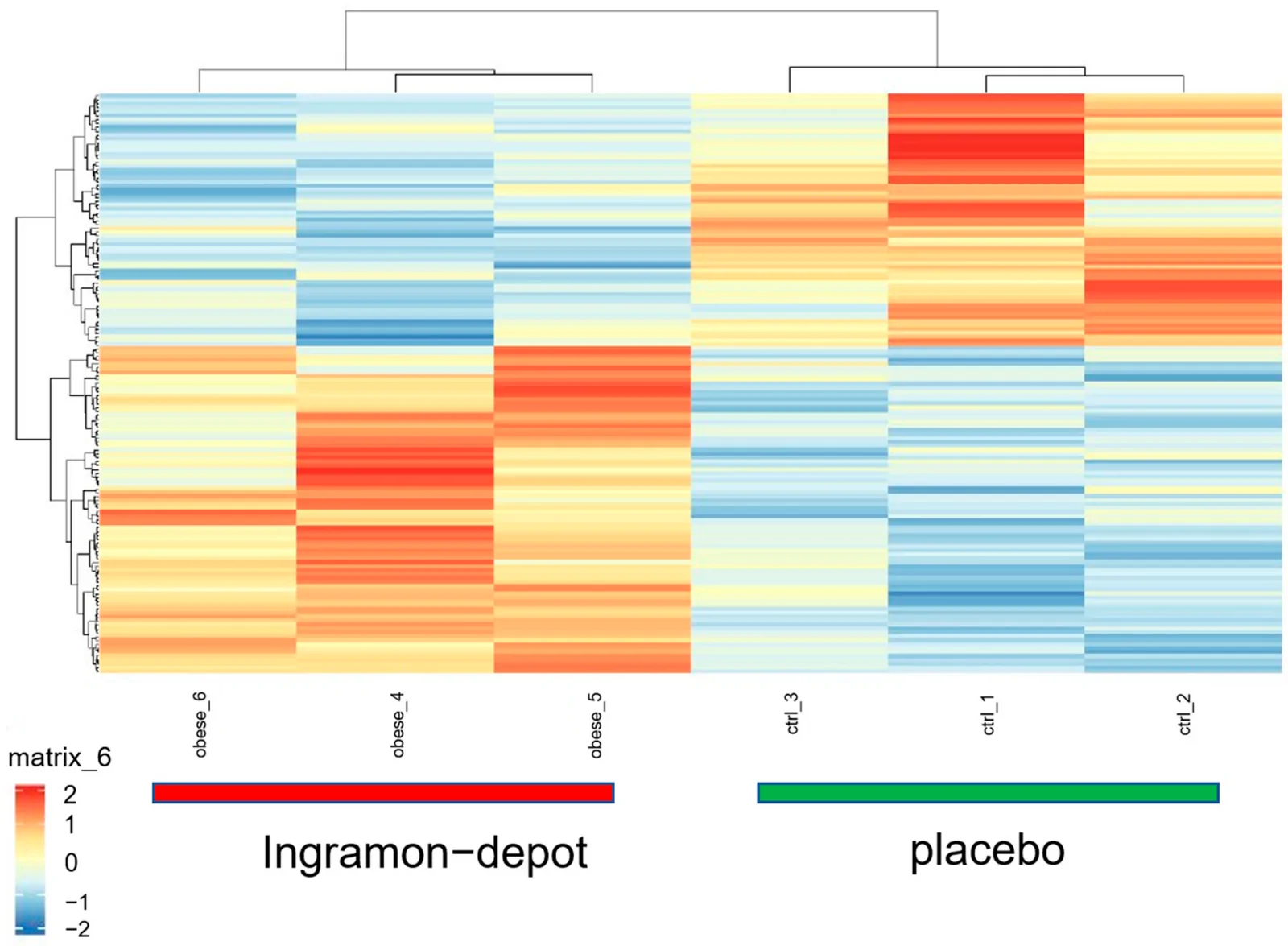
Heat map of myocardial expression of the exploratory gene list (protein-coding genes with nominal *p* < 0.05 and |log2 fold change| > 1; see Figure 8) in db/db mice treated with Ingramon-depot (obese_4–6) or placebo (ctrl_1–3).

**Figure 8:**
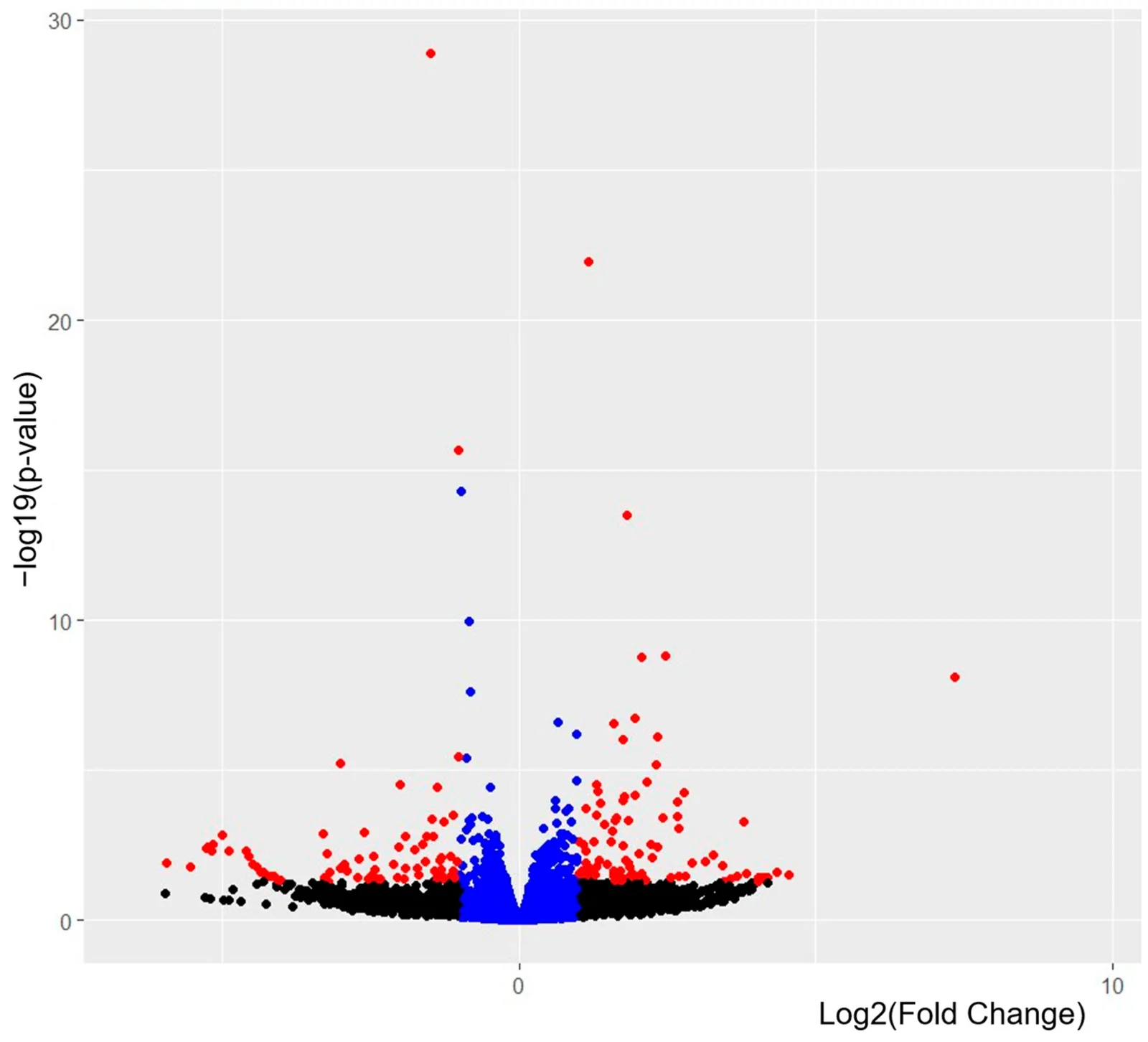
Volcano plot of myocardial gene expression in db/db mice treated with Ingramon-depot or placebo. Each dot represents a gene. Red, genes with nominal *p* < 0.05 and |log2 fold change| > 1; blue, genes with |log2 fold change| ≤ 1; black, genes with |log2 fold change| > 1 and *p* ≥ 0.05. *p*-values are not adjusted for multiple testing; adjusted values are given in Table S2.

**Figure 9:**
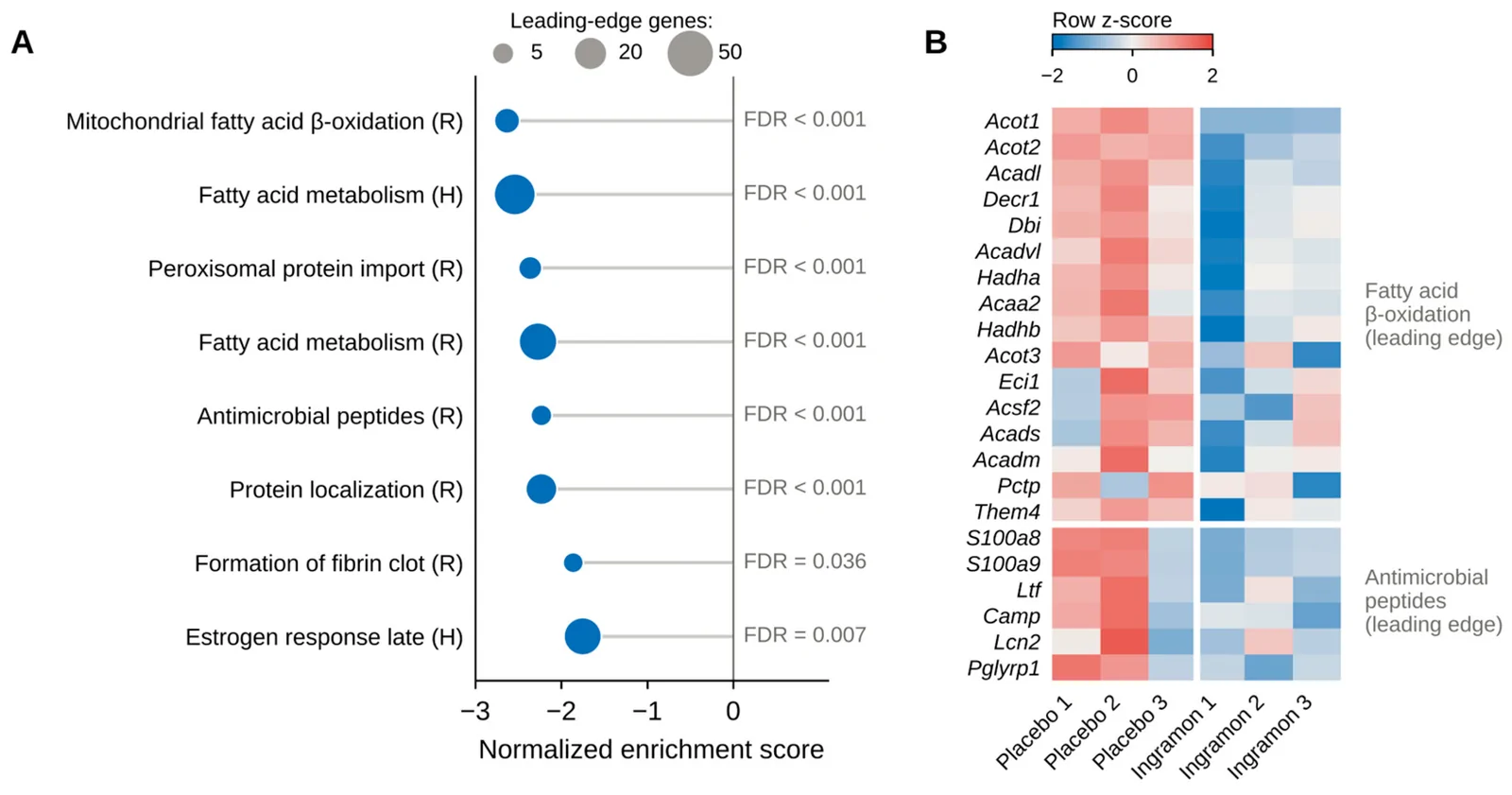
Gene set enrichment analysis of the myocardial transcriptome. (**A**) Gene sets with FDR < 0.05 in pre-ranked GSEA (Reactome (R) and Hallmark (H) collections, MSigDB mouse v2026.1) that remained nominally significant (*p* < 0.05) after exclusion of any single animal. Negative normalized enrichment scores indicate lower expression in the Ingramon-depot group; dot size shows the number of leading-edge genes. No gene set with higher expression in the Ingramon-depot group met these criteria. Complete results are given in Table S3. (**B**) Expression of the leading-edge genes of the Reactome sets “Mitochondrial fatty acid β-oxidation” (all 16 genes) and “Antimicrobial peptides” (the six genes with the largest fold changes) in individual animals; values are row z-scores of log2(normalized counts + 1).

For transcriptomic profiling, mRNA sequencing of the myocardium (heart apex) was performed. At a nominal threshold (*p* < 0.05, |log2 fold change| > 1), 48 genes showed lower and 64 genes showed higher expression in the Ingramon-depot group (Figure 7 and Figure 8). After Benjamini–Hochberg correction, 30 genes remained significant (adjusted *p* < 0.05; Table S2, Supplement). The most pronounced change was lower expression of Hmgcs2 (adjusted *p* = 2 × 10^−25^) together with Acot1 and Acot2, PPARα target genes involved in ketogenesis and fatty acid metabolism. Genes with higher expression included mesothelial markers (Msln, Upk3b, Upk1b, Krt7, Krt8, Lrrn4, Muc16), which may reflect differences in the amount of epicardium in the apical samples. Gene set enrichment analysis showed lower expression of mitochondrial fatty acid β-oxidation genes (NES = −2.63, FDR < 0.001) and of neutrophil granule genes (Reactome “Antimicrobial peptides”, NES = −2.23, FDR < 0.001; leading-edge genes S100a8, S100a9, Camp, Ltf, Lcn2) in the Ingramon-depot group (Figure 9; Table S3, Supplement). Among individual neutrophil-associated genes, only Retnlg remained significant after correction (adjusted *p* = 0.023), whereas S100a8, S100a9, Mmp8, Cd177 and Cxcr2 showed nominal differences only. Broad inflammatory gene sets were not changed (Hallmark “Inflammatory response”, FDR = 0.25).

Thus, Ingramon-depot administration was associated with a shift in adipose tissue leukocyte composition, reflected by a lower F4/80-macrophages-to-CD3+ T-cells ratio. Morphological analysis further demonstrated reductions in myocardial fibrosis and cardiomyocyte degenerative/necrotic changes. Transcriptomic analysis showed lower expression of fatty acid oxidation genes and, at the gene-set level, of neutrophil granule genes in the myocardium of treated mice. However, Ingramon-depot did not affect peripheral blood leukocyte composition or echocardiographic parameters.

## 4. Discussion

Myocardial fibrosis is a major driver of chronic HF progression, cardiac dysfunction, and mortality. In HF with preserved ejection fraction (HFpEF), diffuse interstitial and perivascular fibrosis is predominantly associated with systemic low-grade inflammation linked to cardiometabolic syndrome. Consistent with this concept, bone marrow transplantation from mice with HF induced spontaneous cardiac dysfunction and fibrosis in recipient mice [32]. In this context, sodium–glucose cotransporter 2 inhibitors and glucagon-like peptide-1 agonists are promising therapies because they improve clinical status and exert an anti-fibrotic effect and anti-inflammatory activity [33,34,35]. For example, dapagliflozin prevented adverse cardiac remodeling in sodium–glucose cotransporter 2 global-knockout mice by directly suppressing the macrophage-mediated inflammation and cardiac fibroblast activation [36]. Similarly, semaglutide reduced the epicardial adipose tissue volume index and interstitial myocardial fibrosis in C57BL/6 mice fed a high-fat diet [37]. In addition, the anti-inflammatory, antihypertrophic, antifibrotic, and antioxidant effects of statins may be beneficial in HFpEF [38]. Supporting this possibility, Song X. et al. [39] demonstrated that atorvastatin attenuated cardiac dysfunction by reducing myocardial fiber disruption and interstitial collagen deposition in db/db mice. Taken together, these observations underscore the therapeutic relevance of targeting inflammation-associated myocardial fibrosis in HFpEF. Therefore, in the present study, we investigated the effects of a peptide-based anti-inflammatory agent that inhibits monocyte and neutrophil migration in genetically obese db/db mice.

As an initial step, we characterized the state of the myocardium and assessed immune and biochemical parameters (blood glucose and cardiac troponin I levels) in 16-week-old female db/db mice. Based on published evidence [40] and our previously obtained data [41] indicating that female db/db mice are more susceptible to obesity, and considering the greater similarity to cardiometabolic human HFpEF, female db/db mice were selected for the present study. The mice exhibited hyperglycemia and glycogen deposition in cardiomyocytes and hepatocytes, along with severe obesity. Histological examination further confirmed cardiomyocyte injury, including hypertrophy, dystrophy, granular degeneration/coagulative necrosis, vacuolar (fatty) degeneration, hyaline degeneration, nuclear alterations, and diffuse myocardial fibrosis. Compared with control C57BL/6 mice with normal values [42], transthoracic echocardiography revealed impaired LV relaxation without structural abnormalities. However, a more detailed assessment of LV diastolic function was not possible because of limitations of the ultrasound equipment. Notably, these findings are consistent with those of Lin K. et al. [43], who reported that echocardiography did not detect early-stage cardiac dysfunction in 12-week-old db/db mice, whereas positron emission tomography/computer tomography imaging revealed increased myocardial radiotracer uptake in both early- and late-stage diabetic mice. Additionally, Zhang H-K et al. [44] found that the E′/A′ and E/e′ ratios were the most sensitive parameters for indicating LV diastolic dysfunction, while no significant differences were observed in the E-wave or E/A ratios between C57BL/6 mice with type 1 or 2 diabetes and a control group. In parallel, no significant cardiac hypertrophy was observed, as LVMM was comparable between 16-week-old db/db mice and control C57BL/6 mice. A modest increase in LVMM was observed in 32-week-old db/db mice, irrespective of treatment with Ingramon-depot or placebo. This finding is biologically plausible because, in this mouse model, LV hypertrophy does not occur in the absence of hypertension induced by vasopressor agents [45]. Moreover, higher peripheral blood neutrophil and monocyte counts in db/db mice than in age- and sex-matched controls indicated systemic chronic inflammation. Overall, these findings confirm that this mouse strain reproduces cardiometabolic alterations characteristic of human disease and are consistent with previous reports [12].

Having established the baseline cardiometabolic phenotype, we next evaluated the effects of Ingramon-depot on local and systemic inflammation and myocardial remodeling in db/db mice. Body weight at 32 weeks did not differ significantly from that at 16 weeks, irrespective of treatment. Likewise, transthoracic echocardiography showed no significant changes in cardiac structure or function after Ingramon-depot treatment. Nevertheless, treated mice exhibited smaller areas of coagulative necrosis and fibrosis than control mice. In addition, BNP concentrations were lower in the Ingramon-depot group than in the placebo group, although the difference was not statistically significant, suggesting a possible reduction in hemodynamic stress on cardiomyocytes, whereas cardiac troponin I levels remained unchanged, providing no evidence of acute myocardial injury. Collectively, these complementary findings are consistent with a potential cardioprotective effect of Ingramon-depot, despite the absence of detectable echocardiographic changes.

A potential mechanistic explanation for these findings is that Ingramon inhibits leukocyte migration to inflammatory sites without affecting leukocyte production in the bone marrow or the composition of circulating leukocytes, which may explain the absence of changes in peripheral blood leukocyte composition [20]. Studies in rodents and primates demonstrated that intramuscular or intravenous administration of Ingramon inhibited monocyte and neutrophil migration to local inflammatory sites [20]. This anti-inflammatory effect was also observed in clinical studies, in which Ingramon administration before and after coronary artery stenting prevented increases in C-reactive protein and fibrinogen concentrations [23,46]. On the basis of these findings, we hypothesize that Ingramon may exert cardioprotective effects by inhibiting monocyte and neutrophil activation and migration into the myocardium and by attenuating systemic inflammation. Consistent with this mechanism, its anti-inflammatory activity was further supported by changes in adipose-tissue leukocyte composition, including a reduced proportion of F4/80-positive macrophages and a shift in the macrophage-to-T-cell ratio. Furthermore, transcriptomic analysis of LV myocardium indicated a lower expression of neutrophil granule genes in the myocardial tissue at the gene-set level. Together, these observations suggest that Ingramon modulates inflammatory-cell recruitment rather than bone marrow leukocyte production.

To place these results in a broader immunological context, monocytes and macrophages have traditionally been considered the principal immune-cell populations involved in myocardial remodeling during chronic inflammation and hyperglycemia. In support of this view, Song et al. [39] reported an increase in pro-inflammatory M1 macrophages (iNOS+) and a decrease in anti-inflammatory M2 macrophages (CD206+) in db/db mice. Similarly, in diet-induced obesity, macrophages infiltrated the myocardium, and high concentrations of pro-inflammatory cytokines, including IL-6 and TNF, were detected in plasma and cardiac tissue homogenates [47]. Single-cell RNA sequencing confirmed a pro-inflammatory profile in ApoE-knockout mice fed a Western diet; these mice also exhibited impaired diastolic function, reduced exercise tolerance, and increased pulmonary congestion [48]. Extending these observations, Gutierrez et al. characterized cardiac macrophage profiles in C57BL/6N mice fed a high-fat diet and given L-NAME in drinking water [49]. Pro-inflammatory CCR2+ MHCII+ macrophages were increased in asymptomatic pre-HFpEF mice at 5 weeks, and their abundance correlated with myocardial fibrosis severity. Importantly, these macrophages remained elevated in mice with established HFpEF at 15 weeks [49]. In this process, as major sources of TNF, IL-1β, IL-6, and transforming growth factor-β, macrophages promote myofibroblast differentiation and collagen synthesis [50]. Thus, substantial evidence supports a central role for macrophage-driven inflammation in myocardial fibrotic remodeling.

In contrast to the well-established role of macrophages, the contribution of neutrophils to myocardial fibrosis remains incompletely understood. This uncertainty partly reflects their short lifespan and rapid death at inflammatory sites, which make neutrophil accumulation difficult to detect during sterile myocardial inflammation. Nevertheless, emerging evidence indicates that neutrophils may contribute to fibrosis [51]. For instance, in a mouse model of transverse aortic constriction-induced myocardial hypertrophy with myeloid-cell Wnt5a deficiency, neutrophil depletion with anti-Ly6G antibodies suppressed inflammatory myocardial infiltration and attenuated cardiomyocyte hypertrophy [52]. Consistent with this observation, myeloperoxidase-deficient mice were resistant to angiotensin II-induced atrial fibrosis [53]. Moreover, mice deficient in peptidyl–arginine deiminase 4, an enzyme involved in histone citrullination during neutrophil extracellular trap (NET) formation, showed no evidence of myocardial remodeling, whereas control animals fed a high-fat diet exhibited typical remodeling [54]. From a mechanistic perspective, during chronic inflammation, neutrophils may accumulate in myocardial capillaries, obstruct blood flow, injure endothelial cells, and impair nutrient delivery to cardiomyocytes. Neutrophil-derived myeloperoxidase and reactive oxygen species reduce nitric oxide bioavailability, suppress protein kinase G activity, and impair cardiomyocyte relaxation, thereby increasing myocardial stiffness. In parallel, NETosis products may act as damage-associated molecular patterns that amplify immune-inflammatory signaling through pattern-recognition receptors [55]. In line with these mechanisms, our previous clinical data indicate that HFpEF is associated with increased numbers of circulating activated neutrophils, suggesting a contribution to myocardial fibrosis [56]. Of particular relevance to diabetes, hyperglycemia markedly enhances NET formation; high glucose concentrations increase NET formation in vitro in neutrophils isolated from healthy volunteers [57,58]. Furthermore, Schommer N. et al. [55] reported an increased myocardial NET burden in patients with HF and diabetes. In mice with streptozotocin-induced diabetes, enhanced neutrophil NETosis was associated with impaired cardiac function and myocardial and renal fibrosis [59]. Taken together, these findings provide a coherent rationale for considering neutrophil activation and NETosis as contributors to diabetic myocardial remodeling.

The transcriptomic data are compatible with, but do not establish, a lower neutrophil content in the myocardium of Ingramon-depot-treated mice: neutrophil granule genes were reduced at the gene-set level, whereas individual genes other than Retnlg did not pass correction for multiple testing, and broad inflammatory gene sets were unchanged. Bulk RNA-seq cannot distinguish fewer neutrophils in the tissue from lower activation per cell. Whether reduced neutrophil accumulation contributes to the smaller areas of necrosis and fibrosis therefore remains a hypothesis that requires confirmation.

## 5. Study Limitations

Despite the promising findings, several methodological considerations should be acknowledged, as they may influence the interpretation and generalizability of the present results. First, the peripheral blood leukocyte analysis did not include all major populations of myeloid and lymphoid cells, which limits the characterization of systemic immune alterations. Second, absolute leukocyte numbers per gram of adipose tissue were not determined; therefore, changes in adipose tissue immune-cell composition should be interpreted primarily as relative differences. Third, LV diastolic function could not be assessed using tissue Doppler imaging, and myocardial strain analysis was not performed because of equipment-related limitations. Fourth, immunohistochemical validation was not included, which limits cell-specific interpretation of the histological and transcriptomic findings. Finally, transcriptomic profiling was performed in three pooled samples per group, without a non-diabetic control group and without independent validation by qRT-PCR or immunohistochemistry; the transcriptomic findings should therefore be regarded as hypothesis-generating.

## 6. Conclusions

In conclusion, the present findings suggest that the peptide Ingramon may exert anti-inflammatory and cardioprotective effects in a murine model of diabetic cardiomyopathy. Its anti-inflammatory activity was associated with altered adipose-tissue leukocyte composition, characterized by a reduced macrophage-to-T-cell ratio, with trends toward lower proportions of F4/80+ macrophages and higher proportions of CD3+ T-cells, and with lower myocardial expression of neutrophil granule genes at the gene-set level. Ingramon-depot treatment was also associated with reduced myocardial necrotic changes and fibrosis. Circulating brain natriuretic peptide concentrations were slightly lower in treated mice, although the difference was not statistically significant. The mechanisms underlying these effects, including a possible influence on inflammatory-cell recruitment, require further investigation.

## Figures and Tables

**Table 1:** Peripheral blood leukocyte profiles in 16-week-old db/db and C57BL/6 mice.

Parameter	16-Week-Old db/db (n = 4)	16-Week-Old C57BL/6 Mice (n = 5)	*p*
Leukocytes, 10^9^/L	4.7 (4.5; 4.9)	4.6 (4.4; 4.9)	0.327
CD115+ monocytes, 10^9^/L	1.42 (0.89; 1.51)	0.15 (0.14; 0.17)	0.014
Ly6G+ neutrophilic granulocytes, 10^9^/L	1.09 (1.05; 1.55)	0.58 (0.44; 0.74)	0.014
Lymphocytes, 10^9^/L	2.5 (1.7; 2.7)	3.5 (3.4; 3.7)	0.014
CD3+ T-cells, 1000/mL	635.3 (475.5; 698.8)	1852.0 (1740.5; 2051.7)	0.014
CD3+CD4+ T-cells, 1000/mL	336.3 (247.4; 377.5)	1000.1 (973.0; 1116.3)	0.014
CD3+CD8+ T-cells, 1000/mL	299.0 (228.2; 312.9)	852.0 (767.6; 935.5)	0.014
CD3+CD4+CD25+ T-cells, 1000/mL	63.4 (51.0; 77.6)	71.3 (70.0; 75.2)	0.624

Data are presented as median (25%; 75%).

**Table 2:** Echocardiographic structural and functional cardiac parameters in 16-week-old db/db and C57BL/6 mice.

Parameter	16-Week-Old db/db (n = 4)	16-Week-Old C57BL/6 Mice (n = 5)	*p*-Value
LA size, mm	2.4 (2.1; 2.7)	2.1 (2.0; 2.4)	0.184
LVIDd, mm	4.5 (4.2; 4.8)	4.3 (4.2; 4.6)	0.591
LVIDs, mm	2.7 (2.6; 3.1)	3.1 (3.0; 3.3)	0.587
LV EDV, mL	92.7 (78.6; 110.3)	85.4 (78.6; 97.3)	0.591
LV ESV, mL	36.5 (31.1; 42.7)	39.4 (35.0; 44.1)	0.587
LV EF, %	61.4 (56.4; 65.2)	56.1 (54.6; 57.8)	0.135
LV SF, %	33.0 (29.4; 35.6)	28.6 (26.5; 30.3)	0.135
LV MM, mg	87.6 (81.6; 93.6)	84.1 (82.2; 87.2)	0.394
RWT	0.24 (0.21; 0.27)	0.24 (0.23; 0.27)	0.831
Mitral E/A ratio	1.6 (1.3; 1.9)	2.0 (1.9; 2.2)	0.021
Tei index	0.63 (0.43; 0.71)	0.67 (0.54; 0.77)	0.134

Data are presented as median (25%; 75%). Abbreviations: EDV—end-diastolic volume; EF—ejection fraction; ESV—end-systolic volume; LA—left atrium; LV—left ventricle; LVIDd—end-diastolic left ventricular internal diameter; LVIDs—end-systolic left ventricular internal diameter; MM—myocardial mass; RWT—relative wall thickness; SF—shortening fraction.

**Table 3:** Peripheral blood leukocyte profiles in 32-week-old db/db mice treated with Ingramon-depot or placebo.

Parameter	32-Week-Old db/db Ingramon-Depot (n = 13)	32-Week-Old db/db Placebo (n = 11)	*p*-Value
Leukocytes, 10^9^/L	4.9 (3.5; 5.4)	5.7 (4.7; 6.2)	0.465
CD115+ monocytes, 10^9^/L	1.1 (0.7; 1.2)	1.4 (0.9; 1.5)	0.537
Ly6G+ neutrophilic granulocytes, 10^9^/L	1.1 (0.9; 1.2)	1.1 (1.1; 1.5)	0.584
Lymphocytes, 10^9^/L	1.8 (1.4; 2.2)	2.5 (1.7; 2.7)	0.201
CD3+ T-cells, 1000/mL	511.7 (429.3; 656.1)	635.3 (539.9; 691.6)	0.792
CD3+CD4+ T-cells, 1000/mL	267.6 (212.2; 381.8)	336.3 (247.3; 377.5)	0.715
CD3+CD8+ T-cells, 1000/mL	244.11 (207.1; 284.4)	298.97 (263.87; 308.7)	0.428
CD3+CD4+CD25+ T-cells, 1000/mL	54.3 (38.3; 67.0)	63.5 (51.0; 77.6)	0.428

Data are presented as median (25%; 75%).

**Table 4:** Adipose tissue leukocytes in 32-week-old db/db mice treated with Ingramon-depot or placebo.

Parameter	32-Week-Old db/db Ingramon-Depot (n = 13)	32-Week-Old db/db Placebo (n = 11)	*p*-Value
F4/80+ cells, % CD45	21.0 (8.3; 50.0)	59.0 (27.8; 60.7)	0.128
F4/80+CD206+ cells, % CD45	6.0 (2.2; 27.7)	26.0 (13.1; 33.0)	0.052
F4/80+CD206- cells, % CD45	13.0 (6.5; 22.3)	26.0 (14.7; 30.7)	0.171
Ly6G+ cells, %CD45	7.0 (5.4; 12.5)	8.0 (5.0; 10.5)	0.967
CD3+ T-cells, %CD45	30.0 (28.3; 51.3)	15.0 (11.5; 22.7)	0.120
F4/80 / CD3+ T-cell ratio	0.8 (0.2; 2.5)	2.9 (1.1; 4.7)	0.037

Data are presented as median (25%; 75%).

**Table 5:** Echocardiographic structural and functional cardiac parameters in 32-week-old db/db mice treated with Ingramon-depot or placebo.

Parameter	32-Week-Old db/db Ingramon-Depot (n = 13)	32-Week-Old db/db Placebo (n = 11)	*p*-Value
LA size, mm	2.5 (2.4; 3.0)	2.4 (2.3; 2.8)	0.520
LVIDd, mm	4.2 (4.1; 4.6)	4.2 (4.1; 4.5)	0.630
LVIDs, mm	2.6 (2.6; 3.1)	2.7 (2.5; 3.1)	1.000
LV EDV, mL	79.7 (76.4; 97.3)	76.4 (74.2; 92.5)	0.630
LV ESV, mL	25.8 (24.6; 38.0)	28.2 (22.3; 38.0)	1.000
LV EF, %	63.6 (63.0; 67.8)	63.0 (59.0; 68.1)	0.631
LV SF, %	34.5 (33.3; 37.5)	33.7 (31.1; 37.5)	0.688
LV MM, mg	111.2 (104.5; 154.1)	103.2(97.6; 115.5)	0.149
RWT	0.35 (0.33; 0.36)	0.33 (0.29; 0.35)	0.423
Mitral E/A ratio	2.0 (1.6; 2.4)	1.9 (1.5; 2.1)	0.806
Tei index	0.57 (0.35; 0.81)	0.87 (0.39; 1.15)	0.096

Data are presented as median (25%; 75%). Abbreviations: EDV—end-diastolic volume; EF—ejection fraction; ESV—end-systolic volume; LA—left atrium; LV—left ventricle; LVIDd—end-diastolic left ventricular internal diameter; LVIDs—end-systolic left ventricular internal diameter; MM—left ventricular myocardial mass; RWT—relative wall thickness; SF—shortening fraction.

## Data Availability

The original contributions presented in this study are included in the article/Supplementary Material. Further inquiries can be directed to the corresponding author.

## Supplementary Materials

The following supporting information can be downloaded at https://www.mdpi.com/article/10.3390/biomedicines14102341/s1, Table S1: Per-sample sequencing depth and read-assignment metrics; Table S2: DESeq2 results for all tested genes, with nominal and Benjamini–Hochberg adjusted *p*-values and normalized counts per animal; Table S3: Pre-ranked gene set enrichment analysis results with the leave-one-animal-out robustness check; Figure S1: A-Flow cytometric gating strategies for blood leukocyte subpopulations: A-T-cell populations (CD3+CD4+, CD3+CD8+, CD3+CD4+CD25+). B-Neutrophilic granulocytes and monocytes; Figure S2: Flow cytometric gating strategies for adipose tissue leukocyte subpopulations: A-T-cells and neutrophilic granulocytes. B-Macrophages.

## Abbreviations

The following abbreviations are used in this manuscript:

BNP	Brain natriuretic peptide
CCL2	C-C motif chemokine ligand 2
CXCL12	C-X-C motif chemokine ligand 12
CCR2	C-C motif chemokine receptor type 2
EDV	End-diastolic volume
ELISA	Enzyme-linked immunosorbent assay
ESV	End-systolic volume
H&E	Hematoxylin–eosin
HF	Heart failure
HFpEF	Heart failure with preserved ejection fraction
IL	Interleukin
IVRT	Isovolumetric relaxation time
IVCT	Isovolumetric contraction time
LV	Left ventricle
LVEF	Left ventricular ejection fraction
LVIDd	End-diastolic left ventricular internal diameter
LVIDs	End-systolic left ventricular internal diameter
LVMM	Left ventricular myocardial mass
MCP-1	Monocytic chemotactic protein-1
MT	Masson trichrome
NET	Neutrophil extracellular trap
PAS	Periodic acid-Schiff
PLGA	Poly(lactic-co-glycolic acid)
PWTd	Posterior left ventricular wall thickness
RWT	Relative wall thickness
SWTd	Septum wall thickness
TNF	Tumor necrosis factor
